# Topical Molecular Iodine: An Optimal Biocide Constrained by Inadequate Formulations

**DOI:** 10.3390/ijms26104853

**Published:** 2025-05-19

**Authors:** Jack Kessler, Sarah E. Hooper

**Affiliations:** 1 I2Pure Incorporated, 44679 Endicott Drive, Ashburn, VA 20147, USA; 2 Microbiology and Infection Research Group, Cardiff School of Health Sciences, Cardiff Metropolitan University, Cardiff CF5 2YB, UK; smaddocks@cardiffmet.ac.uk

**Keywords:** molecular iodine, I2, I2 vapor, human skin, biocompatibility, biofilm

## Abstract

The only biocidal iodine species in topical iodine disinfectants is molecular iodine (I2). I2, a biomolecule, has broad-spectrum antimicrobial activity and does not generate resistance. Physicians, regulatory agencies, and scientists have assumed that I2 is responsible for the skin staining and irritation associated with the clinical use of iodine disinfectants; this assumption is deeply embedded in the medical community but is not supported with empirical data. This study provides the first experimental data that measure the biocompatibility of I2 with human skin. Human skin explants in tissue culture were evaluated at 3, 7, and 24 h after being exposed to I2 (500 to 15,000 ppm). Cell viability was measured relative to phosphate-buffered saline using 3-[4,5-dimethylthiazol-2yl]-2,5-diphenyl-tetrazolium bromide (MTT). The biocidal activity of I2 vapor emitted from silicone was demonstrated against bacteria growing on agar to confirm I2 off-gassing from skin was an active biocide. Additionally, statistically significant bacterial reductions with both gas and solution phase I2 were observed in a static and dynamic five-species wound biofilm. The data suggest that high, e.g., 50–5000 ppm, levels of I2 should be incorporated into topical iodine disinfectants instead of the very low (0.2–10 ppm) levels found in 10% povidone iodine products currently in use.

## 1. Introduction

We previously demonstrated [1] that I2 is not responsible for skin staining and provided evidence that I2 partitions into a hydrophobic lipid environment in the hypodermis which serves to stabilize the molecule for hours after absorption. Recent reports indicate that resident bacteria in pilosebaceous sweat glands are responsible for many post-surgical infections [2,3]. Delivering high concentrations of I2 into the skin may have presurgical clinical utility if safety can be established. The primary objective of this study was to evaluate the cytotoxicity of I2 at elevated concentrations in human skin.

Topical toxicity has been a primary formulation consideration for formulators of iodine disinfectants over the past century. In fact, a popular iodophor (Wescodyne) was labeled and promoted as *Tamed Iodine*. Efforts to mitigate the irritancy of Lugol’s solution and iodine tincture led to the commercial introduction of iodophors [4] that contain low (0.2–10 ppm) concentrations [5] of unbound I2.

In 1954, Carrol examined the behavior of I2 in the presence of iodide and reached the following conclusion [6]: *the literature appears to give no clue as to the relative toxicity of the various inorganic forms of iodine. When such evidence is obtained, a re-evaluation of the potentialities of iodine as a chemotherapeutic agent will be possible*. The chemical properties of molecular iodine (hydrophobic and reactive with biomolecules) in solution are distinct from the properties (charged and non-reactive with biomolecules) of most of the other iodine species found in iodophors, e.g., iodide, tri-iodide, polyiodides, and iodate. Seven decades removed from Carrol’s observations, the unsupported assumptions about I2 topical toxicity are accepted more broadly than the medical myth of iodine allergy [7,8,9]. A clear expression of the ubiquitous presumption that molecular iodine is responsible for irritancy is provided in Wikipedia: *because it contains free iodine, Lugol’s solution at 2% or 5% concentration without dilution is irritating and destructive to mucosa*. However, the limited controlled data that examine the topical toxicity of I2 [1,10] suggest that it is not an irritant.

Consequently, the cytotoxicity of I2 is an important factor to determine since loading this molecule into the skin hypodermis with the object of establishing I2 residency at surgical sites for hours may benefit post-surgical outcomes. This manuscript provides controlled data demonstrating that (1) the I2 species is biocompatible with intact human skin at concentrations >1000 times higher than those found in the typical 10% povidone–iodine composition. In addition, we demonstrate that I2 (liquid and vapor phase) is an active biocide and has strong activity against wound biofilms.

## 2. Results and Discussion

We evaluated several different solvents to use for testing the topical toxicity of I2 with human skin and selected glycerin because (1) glycerin is not characterized as a topical irritant, (2) I2 is stable in glycerin, and (3) glycerin reduces the vapor pressure of I2 [11] which favors partitioning of I2 into skin instead of atmospheric loss.

**Figure 1:**
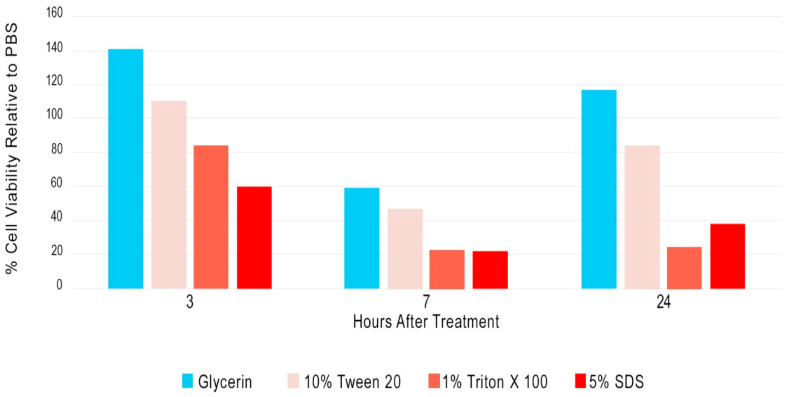
Human skin cell viability of carrier (glycerin) and positive controls. Skin explants were maintained in tissue culture and 3-[4,5-dimethylthiazol-2yl]-2,5-diphenyl-tetrazolium bromide (MTT) reagent was used to evaluate mitochondrial activity. The reported value is the average (n = 3) cell viability normalized to a phosphate buffer (PBS) at each time point. Test agents included the following established human skin irritants (10% Tween 20, 1% Triton X-100, and 5% sodium dodecyl sulfate).

The cell viability of human skin explants was measured using 3-[4,5-dimethylthiazol-2yl]-2,5-diphenyl-tetrazolium bromide (MTT) reagent at three timepoints (3, 7, and 24 h). Figure 1 shows the cell viability of glycerin and three known human skin irritants: 10% Tween 20 (mild irritant), 1% Triton X-100 (moderate irritant), and 5% sodium dodecyl sulfate (SDS; strong irritant). The average value of three samples is reported for each data point and compared to a phosphate-buffered saline (PBS) control at each timepoint. PBS controls are assigned a 100% cell viability value. The reported cell viability value for glycerin was above 50% of their respective PBS control values at all time points. Variability in the glycerin/PBS value was exhibited among the different exposure times; however, the expected relative relationship for cell viability among the mild (Tween 20) versus stronger irritants (Triton & SDS) was observed at all time points, and the 24 h glycerin value was more than 80% of that for PBS. The data indicate that glycerin is a suitable organic carrier to evaluate the biocompatibility of I2 with human skin.

**Figure 2:**
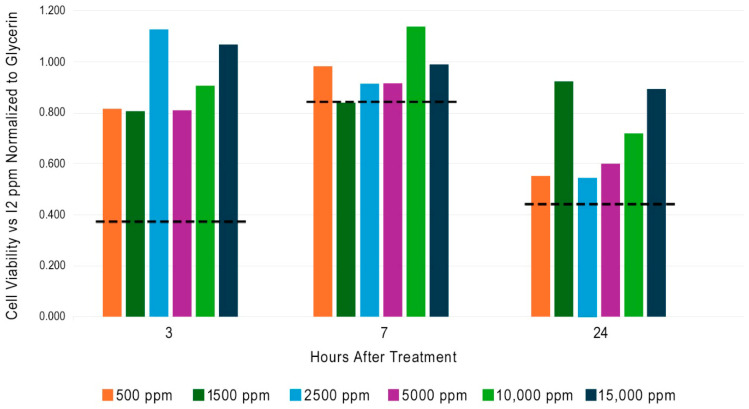
Human skin cell viability versus I2 concentration. I2 (500 to 15,000 ppm) in glycerin. Skin explants were maintained in tissue culture and 3-[4,5-dimethylthiazol-2yl]-2,5-diphenyl-tetrazolium bromide (MTT) reagent was used to evaluate mitochondrial activity. The reported value is the average (n = 3) cell viability normalized to a phosphate buffer (PBS) at each time point. The dashed line (---) at each timepoint displays the normalized cell viability value that corresponds to 50% of the PBS control.

Six concentrations of I2 (500 to 15,000 ppm) in glycerin were tested on human skin explants using MTT reagent. The observed values at each timepoint were normalized to their respective glycerin control and are shown in Figure 2. The dashed line in Figure 2 displays the normalized cell viability value that corresponds to 50% of the PBS control at the respective time point. All human skin explants treated with I2 are higher than 50% of the viability obtained with PBS. Perhaps the strongest aspect of this data set is the absence of a decrease in cell viability in response to increased I2 concentration at any of the three timepoints. Figure 2 demonstrates that I2 is biocompatible with skin at concentrations that are more than >1000 times higher than those found in a typical 10% PVP-I composition. This result may seem surprising given the dogma of I2 toxicity found in the medical literature, but we should remember that controlled measurements with pure I2 have not been previously conducted.

**Figure 3:**
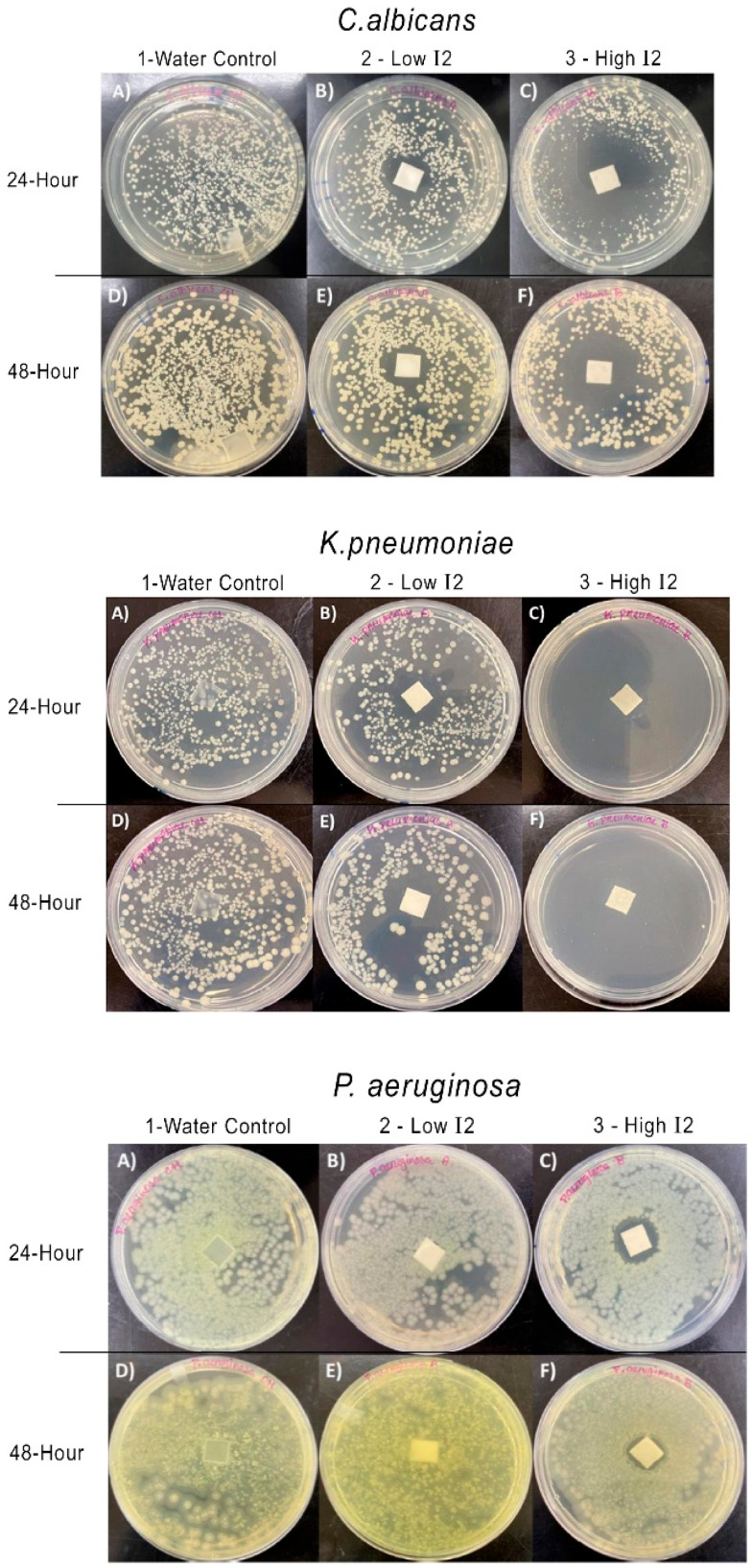
Growth of *C. albicans*, *K. pneumoniae*, and *P. aeruginosa* with and without the presence of I2 gas. Plate images shown at 24 and 48 h in the absence (**A**,**D**) or presence (**B**,**C**,**E**,**F**) of I2 gas at low (**B**,**E**) or high (**C**,**F**) I2 concentrations. Silicone coupons were treated with 300 ppm aqueous I2 ((**B**,**E**) low I2) or 15,000 ppm I2 in glycerin ((**C**,**F**) high I2).

We evaluated the antimicrobial activity of I2 vapor by impregnating I2 into 1 cm coupons of medical grade silicon and observing their effect on microbial growth on seeded media. Figure 3 shows microbial growth at baseline and at 24 and 48 h for three of the seven organisms evaluated. In each set of six images, the first column (A and D) corresponds to the negative control (water treatment); the 2nd column (B and E) corresponds to coupons treated with 300 ppm aqueous I2, and the third column (C and F) corresponds to coupons treated with 15,000 ppm I2-glycerin. The 1st row corresponds to 24 h growth, and the 2nd row shows 48 h growth at 37 ± 2 °C in 5% CO2. Images are shown for (I) *C. albicans* ATCC 90028, (II) *K. pneumoniae* ATCC 700603, and (III) *P. aeruginosa* ATCC 15442. Similar results were obtained with *E. faecalis* ATCC 51299, *E. faecium* ATCC 700221, *S. epidermidis* ATCC 51625, and *P. mirabilis* ATCC 29245.

I2 gas emitted from treated silicone coupons exhibited antimicrobial activity which is consistent with prior reports in the literature [12,13,14]. Microbial sensitivity to I2 vapor differed among organisms, and biocidal activity was concentration dependent. These observations are consistent with those made by Gottardi when he measured a reduction in suspended bacteria contacted with human skin that had been infused with I2.

**Figure 4:**
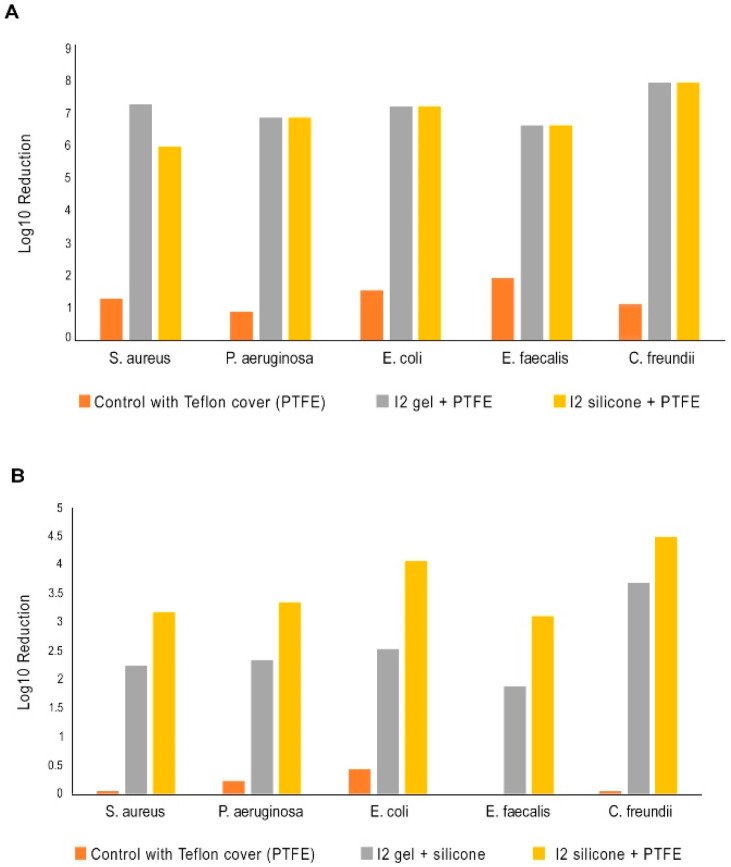
A and 4B. Biocidal efficacy of I2 in a static (**A**) and a dynamic (**B**) 5-species wound biofilm. I2 delivered via a 10,000 ppm gel or as I2 gas (1/16th inch thick silicone treated with 15,000 ppm I2/glycerin located at the top of a well). A thin layer of polytetrafluoroethylene (PTFE) covered the top of wells treated with I2. Bacterial enumeration at 24 h showed that each bacterium was significantly reduced (*p* < 0.05) for both treatments under static and flow conditions.

Figure 4 shows the biocidal effectiveness of I2 gas in a static and a dynamic five-species wound biofilm; statistically significant log reductions are indicated with an asterisk. Biofilms were prepared on agarose–collagen plugs that were grown in a 24-well plate as described in the Materials and Methods section below. A gel containing 13,000 ppm I2 was used as positive control. Medical grade silicone 1/16th inch thick was impregnated with 16,000 pm I2-glycerin and 8 mm circular sections were prepared using a punch. A total of 0.15 g of the I_2_ gel (13,000 ppm I2) or a single I_2_-impregnated piece of silicone (16,000 ppm) was applied to the surface of each matrix to simulate the application of a topical treatment to a wound. In each instance, the well was overlaid with an 8 mm disk of polytetrafluoroethylene (PTFE). Equivalent untreated biofilms and vehicle control biofilms were also overlaid with an 8 mm disk of PTFE. Biofilms were collected at 24 h and homogenized in 1 mL phosphate-buffered saline (PBS) using a sterile 2 mL glass homogenizer. Bacteria were enumerated 24 h post-treatment. In the static biofilm model, both the I2-gel and I2-treated silicon coupon demonstrated a 6–8 log bacterial reduction which was statistically significantly (*p* < 0.05) as compared to controls (1–2 log bacterial reduction). The dynamic biofilm model better represents the real-world wound environment, and a single exposure to the I2-gel or the I2-silicon coupon also demonstrated a statistically significant (*p* < 0.05) reduction in all bacteria as compared to controls.

## 3. Materials and Methods

### 3.1. Ex Vivo Human Skin Explant Cell Viability

Adipose tissue was removed from human skin tissue that was procured from a post-surgical procedure under an IRB-exempt protocol. Intact human skin tissue explants (12 mm) were produced via biopsy punch and trimmed to an even thickness. Punched explants were transferred to the insert of a 6-well Corning Transwell cell culture plate and one mL of Roswell Park Memorial Institute (RPMI) 1640 medium (ATCC) that contained 2% penicillin/streptomycin was placed in each well below the Transwell insert. Experimental treatments (10 μL) were placed on the surface of the explants and spread evenly across the surface using a sterile inoculation loop to ensure even coverage without allowing any material to go over the sides of the explant. Explants were then incubated at 37 °C for 3, 7, or 24 h. Cell viability was evaluated (n = 3 for each timepoint/treatment) using 3-[4,5-dimethylthiazol-2yl]-2,5-diphenyl-tetrazolium bromide (MTT) reagent from R&D Systems (Minneapolis, MN, USA). Conversion of MTT into formazan was measured at 570 nm on a microtiter plate reader. Reported readings at each time point were normalized to the absorbance read with phosphate-buffered saline (PBS) measured at that time point. PBS and glycerin (solvent used for I2 test articles) were used as negative controls. Tween 20 (Boston BioProducts Inc., Milford, MA, USA) 10% (*v*/*v*) was used as mild positive irritant control; Triton ×100 (Millipore-Sigma, St. Louis, MO, USA) 1% (*v*/*v*) was used as a moderate irritant control; and SDS (sodium dodecylsulfate, Biorad Laboratories, Hercules, CA, USA) 5% (*v*/*v*) was used as a strong irritant control.

### 3.2. I_2_ Vapor Phase Antimicrobial Activity with Silicone Coupons

Platinum-cured medical grade silicone squares (1 cm) with a thickness of 0.16 cm (coupons) were stored for two or more days at room temperature in (a) distilled water, (b) 300 ppm I2 in distilled water, or (c) 15,000 ppm I2 in glycerin. Prior to testing, each category of coupon was placed in a petri dish with 25 mL of sterile distilled water and gently swirled 10 times in a clockwise direction and then swirled 10 times in a counterclockwise direction. After the final wash, coupons were transferred onto sterile gauze and dried by patting each sample against the gauze. The coupons were then transferred into a second sterile dish that contained 25 mL of sterile distilled water using sterile metal forceps and flipped over so that their topside was now facing down and swirled as described above. This process was repeated for each test organism.

Test cultures were initiated from frozen glycerol stocks and streaked onto fresh plates of growth media as indicated: *C. albicans* ATCC 90028–Sabouraud Dextrose Agar; *E. faecalis* ATCC 51299–Brain Heart Infusion Agar (BHI); *E. faecium* ATCC 700221–BHI; *K. pneumoniae* ATCC 700603–Nutrient agar; *P. aeruginosa* ATCC 15442–Tryptic Soy Agar; *P. mirabilis* ATCC 29245–Nutrient agar; *S. aureus* ATCC 6538–Mannitol Salt Agar (MSA); and *S. epidermidis* ATCC 51625–MSA. Plates were incubated at 37 ± 2°C and 5% CO2 overnight or until sufficient growth was observed. Single colony isolates were used to inoculate 5 mL of Tryptic Soy Broth (TSB), and the resulting suspensions were incubated with shaking at 37 ± 2 °C for 16–18 h. Following incubation, subcultures were diluted to approximately 1.5 × 10^4^ CFU/mL. For each organism, 100 μL of inoculum was spread across plates such that total recoverable colonies after incubation would be between 300 and 1500 CFUs per plate. One coupon from each test group (water, aqueous I2, and glycerin I2) was placed centrally onto an inoculated plate, and the plates were incubated at 37 ± 2 °C in 5% CO2 for 48 h. Plates were photographed at 24 and 48 h for visual comparison between the treatment groups.

### 3.3. Biofilm Testing

*Staphylococcus aureus* EMRSA-15, *Pseudomonas aeruginosa* ATCC 9027, *Citrobacter freundii* NCTC 6272, *Escherichia coli* ATCC 10418, and *Enterococcus faecalis* ATCC 19433 were maintained on nutrient agar or tryptic soy agar. Selective media for re-isolation from mixed biofilms were UTI Chrome (*E. coli*), Coliform Chrome (*C. freundii*), Slanetz and Bartely (*E. faecalis*), Cetrimide (*P. aeruginosa*), and Baird Parker (*S. aureus*). All media were purchased from Sigma-Aldrich (St. Louis, MO, USA). Each species was cultured in 5 ml Nutrient Broth or Tryptic Soy Broth for 16 h at 37 °C and subsequently equilibrated to 1 × 10^8^ CFU ml-1 in a simulated wound fluid (SWF) base (2.34 mM CaCl2.2H2O, 3.75 mM KCl, and 9.9 mM NaCl) [15]. A 5 mL mixture containing a 1:1 ratio of each species was prepared and used in subsequent experiments.

Preparation of substrata, inoculated matrices, and static and dynamic (flow) models followed the method of Khalid [16]. Agarose–collagen was prepared by adding fetal bovine serum (FBS; heat inactivated; Pan Biotech, Jestetten, Germany) to a 1 mL bacterial suspension to achieve a concentration of 3% *v*/*v*, NaHCO3 was added to achieve a concentration of 100 mM, and bovine skin collagen was added to a concentration of 0.2 mg mL^−1^. Then, 20 mL of 1.5% (*w*/*v*) agarose was dissolved in an SWF base. This was allowed to cool but not set. Then, 10 mL of agarose mixture was poured into the cell suspension. The agarose–bacteria suspension was briefly mixed by inversion, and 780 µL aliquots were dispensed into the wells of a 24-well microtiter plate (MTP; Sigma Aldrich, Gillingham, UK) and set at room temperature before 8 mm agarose plugs were cut from the wells using a sterile leather punch or biopsy punch.

For the static biofilm culture: the biofilms were prepared as described above [16], and inoculated matrices were transferred to a 24-well MTP using sterile forceps. Wells of the plate were filled with 300 mL SWF (SWF base plus 3% *v*/*v* heat-inactivated FBS and 100 mM NaHCO3) [15], which was sufficient to maintain nutrition and a moist environment without covering the surface of the agarose–collagen plug. A lid was fixed to the plate, and the biofilms were incubated at 33 °C. The biofilms were prepared as described above and transferred to the biofilm flow device using sterile forceps. The biofilm flow device was filled with SWF and connected to a peristaltic pump at a flow rate of 0.322 mL min^−1^. The inoculated matrices were transferred into the wells of the flow system and maintained at 33 °C for the duration of the experiment.

For the dynamic biofilm culture: the biofilms were prepared, and the models were set-up as described. Additionally, 0.15 g of a liquid/solid treatment or a single piece of I_2_-impregnated silicone was applied to the surface of each matrix to mimic the application of a wound treatment, and then the top of the well was overlaid with an 8 mm disk of PTFE. Equivalent untreated biofilms were prepared, and vehicle control biofilms were overlaid with an 8 mm disk of PTFE. The biofilms were collected at 24 h and homogenized in 1 ml of phosphate-buffered saline (PBS) using a sterile 2 mL glass homogenizer. Total viable count was measured using the Miles Misra method on selective agars, as indicated previously [17].

### 3.4. Statistical Analysis

The statistical analysis used ANOVA with post hoc Tukey’s test and was undertaken using Minitab Statistical (version 2024) significance was determined as *p* < 0.05. The standard error of the mean was calculated based on three biological and three technical replicates per experiment.

## 4. Conclusions

A systematic description of the non-linear equations that govern the behavior of aqueous iodine chemistry was finally developed almost 2 centuries after the discovery of this element [18]. During this interval, widespread human use of topical iodine compositions led to established medical opinions formed without the benefit of accurate analytical characterization. Researchers have incorrectly assumed that I2 is the irritant in compositions that contain diverse iodine species and failed to consider the complexities of the underlying aqueous chemistry as pointed out by Carroll. The controlled data contained in this manuscript demonstrate that I2 is biocompatible with human skin at concentrations more than 1000 times higher than those found in most commercial 10% PVP-I compositions.

Gottardi’s remarkable observation that I2 outgasses from human skin for more than 8 h [19] after contact is counterintuitive given the reactivity of I2 with biomolecules [20]. Lipids are the only class of biomolecules that could possibly stabilize I2 in mammalian tissue. Additional support for the concept that adipocytes may serve as a reservoir for I2 is found in the observation that the observed 10-fold greater microbicidal activity of iodine versus monochloramine is due to its 1000 fold higher lipophilicity [21]. We believe I2 outgassing from hypodermis tissue occurs because adipocytes serve as a repository for I2. The sequestration and outgassing kinetics of I2 with pig skin hypodermis [1] are consistent with this assumption, and further experimentation could explore the role, if any, of adipocytes in this process. The concept of converting skin into an antimicrobial material is an attractive possibility as Gottardi demonstrated that I2 outgassing inactivates *M. luteus* inoculated onto the skin [19]. However, additional testing will need to evaluate if I2 outgassing will protect the skin from airborne bacteria. Nevertheless, the demonstration of a 3–4 log inactivation of all bacteria species in a five-species biofilm from a single exposure to I2 vapor is intriguing as an I2-emitting material could be incorporated into bandages and used to prevent/treat biofilm formation in wounds.

The biocompatibility of I2 with human skin at concentrations orders of magnitude higher than that found in 10% PVP-I is perhaps not surprising when viewed through an adaptationist lens. For the past 3.5 billion years, peroxidases (POD) protected living systems by reducing reactive oxygen species via electron transfer from halogen anions (iodide/chloride) and/or small (no larger than Pro-Gly-Gly) organic molecules [22,23]. Reaction products (I2 and thyroid hormones) from the peroxidase mediated oxidation of iodide, and tyrosyl groups have been responsible for critical functions in living systems for over 500 years, i.e., living systems have adapted and utilized iodinated biomolecules. The thyroid gland oxidizes about 120 µg of iodide daily in the follicular lumen. The total volume of follicular lumen vesicles is dynamic but is a very small fraction of 1 cubic centimeter (cc). Assuming a 1 cubic centimeter volume for the follicular lumen, a steady-state oxidation of 120 µg of iodide in 1 cc equates to the formation of 5 µg I2/h which is an I2 concentration 1000 times higher than that found in a typical 10% PVP-I product. I2 concentrations at this level ensure rapid iodination of nearby lipids that mediate the Wolff–Chaikoff effect. In fact, surgeons have demonstrated that high iodide levels reduce infarct size in myocardial infarct subjects with blockage of the descending left anterior artery (STEMI) undergoing primary percutaneous coronary intervention [24] which is likely due to iodo-lipid formation after iodide oxidation by MPO.

A topical iodine disinfectant that delivers I2 into skin and establishes a biocidal I2 flux across skin for hours holds the promise of greater medical utility than one that only acts at the surface. In fact, a controlled real-world clinical evaluation of this concept was conducted in the emergency department of Beth Israel Medical Center in New York, NY [25]. The rate of culture contamination from 8467 blood draws over a six-month period was lower (*p* < 0.00001) for skin prepped with iodine tincture (>100 ppm I2) versus 10% PVP-I (2–8 ppm I2). The safety profile for topical I2 suggested by the data in this manuscript indicates that levels of I2 far beyond 100 ppm are feasible for topical application. The I2 molecule has ideal properties: broad antimicrobial activity (viruses, bacteria, fungi, and yeasts); no evidence of resistance; a well-characterized systemic toxicity profile; and anti-inflammatory activity. If physicians are provided with suitably formulated iodine products, they may ultimately demonstrate clinical benefit across a broader range of indications including skin and chronic wound conditions that are difficult to treat.

## Data Availability

The experimental data in this manuscript are unavailable due to privacy restrictions.
